# Concussion of the Brain

**Published:** 1892-09

**Authors:** Sarah N. Smith

**Affiliations:** New York


### CONCUSSION OF THE BRAIN.

SARAH N. SMITH, M. D., NEW YORK.

In December, 1891, one of my patients, living in one of the surburbs of Boston, consulted me relative to a sister, a young lady, some twenty-two years of age, for whom she felt great anxiety. From hints I received, I felt sure that the mind of the sister was impaired from some cause. She could not keep her own wardrobe in order even.

But the sister said there was *one* thing that she could do well, "darn stockings," which is more than some ladies can do.

I learned that she always complained of pain at the base of the brain, extending over the vertex. She never was free from it.

She was not allowed to go into the street alone, and when out with her mother would cling to her like a frightened child.

I elicited the fact that when a child some three years old she fell from the lounge, striking the head on the vertex, causing stupor for a few moments only, then seemed to be as well as ever.

She had no confidence in herself, but was quite unwilling to be dictated to or controlled. She was impatient and peevish, with a vicious temper, when crossed was inclined to be sulky and indolent.

I was informed that they had consulted several allopathic physicians. All agreed that nothing could be done for her. Notwithstanding, I decided to take the case. I felt assured that Homœopathy, *pure* and *simple*, carefully applied was equal to the emergency.

The conditions and symptoms pointed to Arnica.

I sent her mother eight powders of Arnica$^{200}$, Dunham, with instructions to take one powder every other day until four were used, wait one week and take the remaining four in the same way.

When she had used the first four, she told her mother that it seemed so good to be rid of that headache.

For several weeks Arnica was the indicated remedy.

She improved so much from week to week that friends and neighbors asked what had come to Alice.

A few days later her mother was called out of town for the day on business, and cast about to see what disposition could be made of Alice until her return. Alice at once said that if they would order a roast for dinner she could prepare dinner for herself and brother, and she did prepare it with perfect satisfaction to all for the first time in her life, and did it willingly and cheerfully.

Her mother said that she appeared to be *unable* to do what she attempted or what was suggested to her.

To illustrate: She would begin to clear the table after meals; she would stop and hesitate before lifting the thing to be removed; she seemed to lack *will-power* or force to carry out her purpose.

After reviewing the case I attributed this condition to torpor of the nerve centres, *paresis* of the *brain*, which, with other symptoms, suggested Lycopodium. I gave Dunham's 200, with the most satisfactory results. It acted promptly and curatively. It not only brushed away the hesitation and gave her energy of purpose and will-power to carry out that purpose, but her whole nature seemed to be changed. She was no longer fretful, imperious, and uncontrollable, but quiet and submissive in mind and temper. She tries to be kind and helpful and is anxious to improve. So reports her mother.

I wrote her mother at this point that I felt it very necessary for me to see the patient that I might learn just what her greatest needs were. Her mother brought her to New York for a two weeks' stay. I then learned from her mother that when some six or seven years old she had fallen down-stairs, striking her head severely and was unconscious for some time. She did not recover her health entirely. Finally was stricken with blindness for three days.

She complained of great pain in the head, and back of the ear. She was never as well after this second fall. Was very nervous, but attended school, much against the judgment of her teachers. Some weeks, or perhaps months after, when attending school she, without any apparent cause, became blind the second time and continued so for twelve days. She was then taken to an allopathic hospital for treatment. The doctors in attendance pronounced her trouble iritis. They told her friends that she was liable to become permanently blind at any time, unless her general health improved. She was never very strong, but general health fairly good. After the menses appeared, about the age of thirteen, she was somewhat better, but menstruated every three weeks. But from a child of seven years she has complained of the pain in the cerebellum and more or less pain in the spine, without any relief until relieved by the homœopathic remedies that I have given her.

The first day that she arrived in New York she came to my office; said that her head felt very tired, but otherwise she was feeling very well. I gave one powder of Phos.$^{45m}$, F. When she came three days later, she said that her tired head was well.

At her second visit, in three days, I learned that she had a trouble, which she located at the medulla spinalis; said that it hurt her so much when attempting to lie down on a pillow that she always had to put her hand under her head to steady it. "How long have you had *this* pain?" "Always," she replied. I prescribed Apis.$^{200}$, D.'s, and asked her to call in four days. She came at the appointed time; said that her head felt *better* and that last night she laid down "without helping her head." I gave her another powder of Apis.$^{45m}$, F., to be left undisturbed in its action, until I should see her again.

Of course any of these conditions are quite *liable* to return until these lesions and lacerations are thoroughly repaired, and healthy brain tissue established. Light strains may open the wounds afresh and prove fatal to mind and body. She is but a child, mentally, but looks bright and intelligent to all observers, compared with the past. Her family are delighted with the change that Homœopathy has wrought in her and feel confident, as I do, that it will complete the cure. It is their first experience with Homœopathy. The mother and brother were quite opposed to the "new fads," but the sister, who was under my care, insisted upon it, as she had already begun to see the benefit of it in her own case. She also felt as I did, in relation to Alice, that she had very *little* to *lose*, and much to gain.

When Alice goes out with her mother now, she no longer clings to her mother's side, but takes the lead in their journeyings, warning her against dangers, etc.

One could scarcely credit the improvement that this patient made during her two weeks' sojourn in New York city. She seemed to come into a new life, a new world as it were, her slumbering mind awaking to the realities of life, to the enjoyment of her surroundings, and making the most of them.

Does it not seem almost incredible that concussion of the brain, with *such serious consequences*, can be cured and health of body and mind restored, after a lapse of eighteen years?

But Homeopathy has been the gospel of *peace* and *health* to thousands who never dared hope for anything but *pain* and *suffering* till the end, and will be for thousands of others who are still ignorant of *its* power to heal and to save life.

Who dares limit the power of Homœopathy?

